# Hippocampal Glutamate NMDA Receptor Loss Tracks Progression in Alzheimer’s Disease: Quantitative Autoradiography in Postmortem Human Brain

**DOI:** 10.1371/journal.pone.0081244

**Published:** 2013-11-28

**Authors:** Efrat Kravitz, Inna Gaisler-Salomon, Anat Biegon

**Affiliations:** 1 J. Sagol Neuroscience Center, Sheba Medical Center, Ramat Gan, Israel; 2 Department Psychology, University of Haifa, Haifa, Israel; 3 Department Neurology, Stony Brook University School of Medicine, Stony Brook, New York, United States of America; Univ. Kentucky, United States of America

## Abstract

Early Alzheimer's disease (AD) is characterized by memory loss and hippocampal atrophy with relative sparing of basal ganglia. Activation of glutamate NMDA receptors in the hippocampus is an important step in memory formation. We measured the density of NMDA receptors in samples of hippocampus, entorhinal cortex and basal ganglia obtained from subjects who died with pathologically confirmed AD and age- and sex- matched non-demented controls. We found significant decreases in NMDA receptor density in the hippocampus and entorhinal cortex but not in the basal ganglia. Loss of NMDA receptors was significantly correlated with neuropathological progression as assessed by Braak staging postmortem. The same samples were probed for neuroinflammation by measuring the density and gene expression of translocator protein 18kDA (TSPO), an established marker of microglial activation. Unlike NMDA receptor loss, increased densities of TSPO were found in all of the brain regions sampled. However hippocampal, but not striatal TSPO density and gene expression were inversely correlated with NMDA receptor density and positively correlated with Braak stage, suggesting NMDA receptors exacerbate neuroniflammatory damage. The high correlation between hippocampal NMDA receptor loss and disease progression supports the use of non invasive imaging with NMDA receptor tracers and positron emission tomography as a superior method for diagnosis, staging and treatment monitoring of AD *in vivo*.

## Introduction

Despite decades of research, the etiology of sporadic Alzheimer’s disease, a progressive neurodegenerative disorder primarily affecting memory [1] and characterized by progressive deposition of neurofibrillary tangles and amyloid plaques in hippocampal and cortical regions [2], is still unknown. AD currently affects more than 24 million persons globally, and this number is predicted to double by 2040 [3]. The introduction of the PET amyloid imaging agent [^11^C]PIB nearly 2 decades ago [4] raised high hopes for *in vivo* imaging of probable AD as a diagnostic, prognostic and treatment monitoring tool, predicated on the assumption that amyloid imaging would be useful in AD diagnosis, staging and treatment monitoring. Unfortunately, studies in hundreds of demented and non demented subjects have shown that increased [^11^C]PIB uptake in the brain precedes disease onset, is common (>50%) in non-demented elderly subjects [4,5], does not correlate with cognitive decline or atrophy [6,7] and amyloid-antibody-induced reductions in amyloid load detected by [^11^C]PIB did not result in clinical benefit [8]. Therefore, Definite AD diagnosis still needs to be confirmed by postmortem pathological examination and there is an urgent need for new tools for AD diagnosis, prognosis and treatment monitoring.

The neurotransmitter glutamate, acting via N-methyl-D-Aspartate receptors (NMDAR) plays a major role in cognitive processes [9,10]. NMDA-evoked long-term potentiation (LTP) is positively correlated with memory formation and NMDAR antagonists are known to disrupt memory. Postmortem studies have indeed found reduced NMDAR density and gene expression in cortex and hippocampus of AD patients [11,12]; however, there was no attempt to examine other brain regions or to correlate the loss of NMDAR with histopathology, ApoE genotype [13] or other relevant imaging targets such as microgliosis [14,15]. 

Using postmortem samples from several regions of brains from subjects with definite postmortem diagnosis and staging of AD [2], we show here that loss of NMDA receptors in AD matches the anatomy of AD pathology and symptoms and the degree of hippocampal NMDA receptor loss correlates strongly with disease progression (Braak stage, [2]).

## Materials and Methods

### Ethics statement

The work was conducted in Sheba Medical Center in Israel on samples obtained from a tissue bank (http://www.brainbank.nl). Donors or their next of kin gave written informed consent to the Netherlands Brain Bank (NBB) to allow the brain autopsy and to use the material and clinical information for NBB approved research protocols. The protocol was approved by the Sheba Medical Center IRB (4049/06).

### Subjects and samples

Postmortem human frozen samples (~1 g) of hippocampus and striatum from male (*n* = 11) and female (*n* = 12) AD patients and 17 age- and sex-matched controls (9 males and 8 females) were obtained from the Netherlands Brain Bank (NBB), with Braak staging and ApoE genotype when available (Braak stage was not determined for one control subject, and ApoE genotype was not determined for 5 control subjects and 2 AD subjects, table 1). 

**Table 1:** Characteristics of tissue donors.

**Group**	**%women**	**Mean Age ±SD**	**Braak stage**	**n**	**APOE genotype**	**n**
		**(range)**				
**AD**			IV	3	ε3/ε3	6
**(*n* = 23)**	52%	79.7 ± 10.6	V	17	ε4/ε3	12
		(62-94)	VI	3	ε4/ε4	3
					ND	2
**Control**			0	5	ε3/ε3	11
**(*n* = 17)**	47%	79.7 ± 11.7	I	7	ε4/ε3	1
		(56-96)	II	4	ND	5
			ND	1		

ND=not determined

### Sample characteristics and handling

Average postmortem delay (PMD) was 418 ± 242 minutes (522 ± 333 minutes for controls and 341 ± 91 minutes for AD subjects). Similar proportions of AD patients and controls were treated with benzodiazepines and/or opioids, which are routinely administered in the last days of hospitalization. Samples were stored in a -80°C freezer. All frozen samples were cryo-sectioned (Leica cm1850) at -15°C (15 μm) in 25 consecutive series and thaw- mounted onto coated glass slides. The remainder was chopped for homogenate assays. Sections were stored in the -80°C freezer until the time of assay.

### Real-time RT-PCR

For cDNA preparation, brain tissue samples (15-20 mg) were homogenized and RNA was extracted using the Lipid Tissue Mini Kit (Qiagen, Valencia, CA), according to the manufacturer's protocol. RNA concentration and quality were tested using NanoDrop. One microgram RNA was reverse-transcribed using the High-Capacity cDNA Reverse Transcription Kit (Applied Biosystems, Foster City, CA), according to the manufacturer’s protocol.

Quantitative real-time PCR (RT-PCR) was performed with 50 ng of cDNA, using the ABI Prism 7900 device, and the results were analyzed using SDS 2.1 Software (Applied Biosystems). Expression levels of the microgliosis marker TSPO (18kDa translocator protein, [16,17]) and a housekeeping gene (GAPDH) were assessed using specific primers and fluorophore-labeled probes (Assay on Demand; Applied Biosystems). Relative quantification of expression level was performed using the ΔΔC_T_ method [18]. Briefly, for each sample, ΔC_T_ was calculated by (C_T target gene_ - C_T housekeeping gene_). Each plate also contained a (constant) cortical reference sample from our brain repository. ΔΔC_T_ was then calculated for each target gene in each sample by (ΔC_T target sample_ - ΔC_T reference sample,_). Fold change, i.e. change in target expression levels relative to a reference sample, was calculated as 2^-ΔΔC^T. All the reactions were performed in duplicates.

### In vitro quantitative autoradiography

Five consecutive series were used for each autoradiographic assay. Published methodologies were used to label targets on tissue sections [19]. On the day of the assay, 5 cryostat sections were removed from the -80°C freezer and allowed to reach room temperature. The sections were incubated with the ligand of choice at a concentration around the theoretical Kd. Consecutive series were incubated with the radioactive ligands in the absence (total binding, 3 consecutive series) or presence (nonspecific binding, 2 consecutive series) of excess (>1000×Kd) of unlabeled ligand. 

#### [^3^H]MK801 autoradiography

After a 30 minute prewash in 50 mM Tris-acetate buffer (pH 7.4), the sections were incubated for 3 hours at room temperature in 50 mM Tris-acetate buffer at pH 7.4 containing 5 nM [^3^H]MK801 (a selective non-competitive NMDAR antagonist; specific activity 27.5 Ci/mmol, PerkinElmer Life Sciences, Waltham, MA, USA), 30 µM glutamate, and 10 µM glycine. Nonspecific binding was determined in the presence of 10 µM unlabeled MK801. At the end of the incubation, the sections were dipped for 5 seconds in ice-cold buffer and then washed for 90 minutes in cold, fresh buffer, followed by a dip in ice-cold double-distilled water. Sections were dried on a slide warmer at 60°C. Dried sections were apposed to low-energy radiation sensitive film (BioMax MR-1, Kodak) for 8 weeks, alongside commercial calibrated tritium micro scales (Amersham Pharmacia Biotech, Inc., Piscataway, NJ, USA). The films were developed in Kodak D-19, fixed, and dried.

#### [^3^H]PK11195 autoradiography

TSPO autoradiography was performed with [^3^H]PK11195 (a selective TSPO antagonist; specific activity 84.8 Ci/mmol, PerkinElmer Life Sciences, Waltham, MA, USA). Sections were first pre-incubated in phosphate-buffered saline (PBS) for 15 minutes at room temperature, followed by a 30-minute incubation at room temperature with the radioactive ligand. Total binding was determined with 1 nM [^3^H]PK11195. Nonspecific binding was determined on consecutive sections in the presence of excess (20 µM) unlabeled PK11195 (Sigma). Sections were then washed twice for 6 min in 4°C PBS and dipped in 4°C double-distilled water prior to drying to remove buffer salts. Sections were dried on a slide warmer at 60°C. Dried sections were apposed to low-energy radiation sensitive film (BioMax MR-1, Kodak) for 3 weeks, alongside commercial calibrated tritium micro scales (Amersham Pharmacia Biotech, Inc., Piscataway, NJ, USA). The films were developed in Kodak D-19, fixed, and dried.

### Quantitative image analysis

Developed films were scanned using a flatbed scanner (2,400 dpi, 256 gray levels, in neutral conditions - no highlights, shadows, midtones, gamma or sharpen manipulations), digitized and saved in 8-bit Tiff format. Quantitative image analyses are performed using the NIH ImageJ software. Regions of interest (ROIs) in hippocampus samples were identified by an atlas of the human hippocampus [20] and included the cornu ammonis (CA) 1-4 hippocampal fields (pyramidal cell layer), dentate gyrus (DG, granular cell layer), subpyramidal strata of CA1 (SubP, including stratum radiatum, lacunosum, and moleculare), subiculum (Sub) and entorhinal cortex (ECx). ROIs in striatum samples included caudate, putamen and accumbens nucleus combined (Internal capsule white matter excluded). ROIs were drawn on the autoradiograms around the whole extent of each region on all sections where the region could be clearly distinguished on the histologically stained section. Thus the size of the quantitation fields was maximal and proportional to the actual size of the various regions, and included a minimum of 3 measurements per sample. Nonspecific binding was subtracted from total binding to generate specific binding values for statistical analysis.

### Statistical Analysis

All statistical analyses were performed using SPSS 17.0 software. Prior to analysis, normal distribution was confirmed by assessment of skewness and kurtosis values (values between -2 and +2 imply normal distribution) and the Shapiro-Wilk test. We performed analyses for statistically significant differences with the following models: Two-way ANOVA was used for human postmortem assays with diagnosis and sex as fixed-factors. Simple regression, or correlation analyses were used when appropriate, according to the experimental design. Human postmortem analyses were performed separately for each region. Since sex differences were not observed and had no significant interactions with other analyzed factors, data were pooled for both sexes and suitable statistical analyses were applied. When main effects or interactions in the above mentioned analyses were significant, one-way ANOVA or Student’s *t* test were used for *post hoc* analyses. For all Braak stage analyses, if significant combined effect (overall experimental effect) was observed in ANOVA, linear term was examined using the “weighted contrasts” option (adjusted for cell size). Significance level (p<0.05) was determined in 2-tailed tests for all *t* tests.

## Results and Discussion

**Figure 1:**
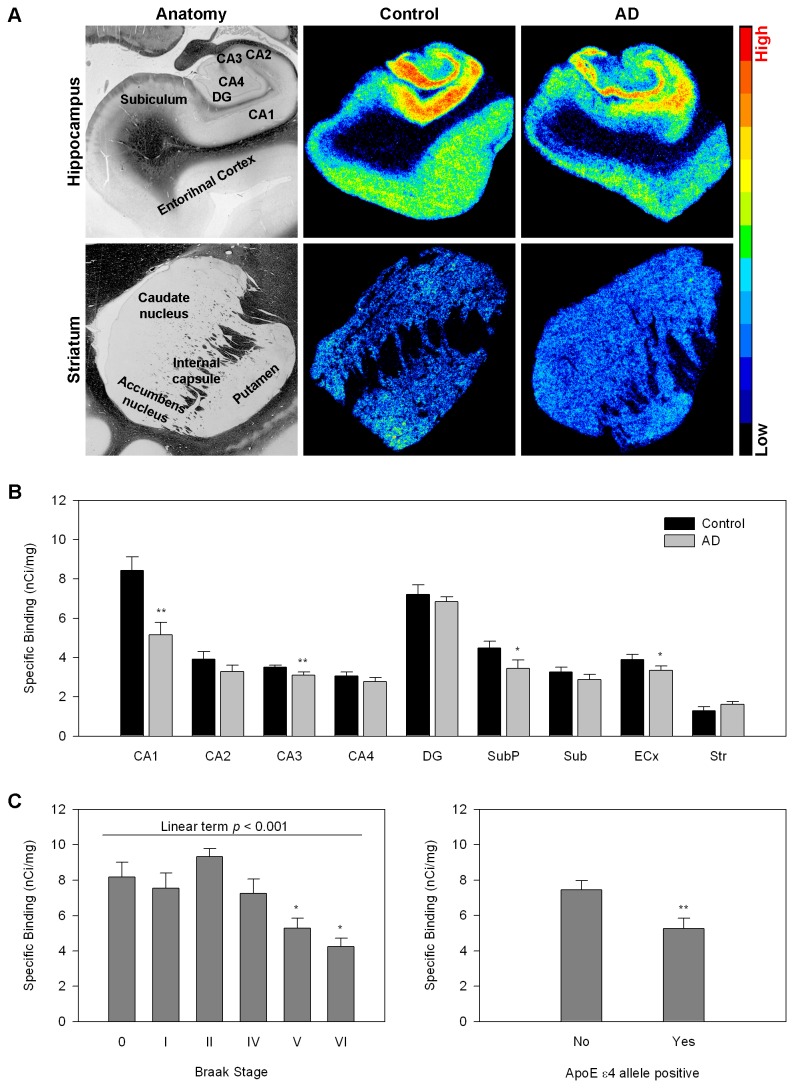
Regional NMDA receptor density in AD and control subjects measured with [^3^H]MK801 autoradiography. A. Representative autoradiographic and anatomical images. Top –Hippocampal anatomy is shown on the left followed by a representative pseudocolored NMDAR autoradiogram from hippocampus of a control subject and a representative autoradiogram from hippocampus of a subject with AD. The bottom row depicts striatal anatomy on the left followed by a representative NMDAR autoradiogram from a control striatum and a representative autoradiogram from a subject with AD. Autoradiograms were pseudocolored using the rainbow spectrum (bar on the right). B. Quantitative autoradiographic measurements of regional NMDAR density (expressed as specific binding of [^3^H]MK801 in nCi/mg tissue). Abbreviations: CA1-4 cornu ammoni fields of the hippocampus, DG=gentate gyrus, SubP=subpyramidal layers of CA1(including stratum radiatum, lacunosum, and moleculare),, Sub=subiculum, ECx=entorhinal cortex, Str=striatum. *p<0.05, **p<0.005 AD relative to control; ANOVA followed by Fisher’s PLSD posthoc test. C. NMDAR density in the CA1 pyramidal layer as a function of Braak stage (left) and genotype (right). *p<0.05 relative to Braak stages 0-II, one way ANOVA followed by Fisher’s PLSD posthoc test. **p<0.005, student’s t test.

Employing quantitative in vitro autoradiography with the specific NMDAR antagonist [^3^H]MK801, we have examined the regional density of NMDAR in the hippocampal formation, entorhinal cortex and basal ganglia of subjects (N=23) who died with AD and 17 age- and sex matched non-demented controls (Table 1). We found significant decreases in NMDAR in hippocampal fields and in the entorhinal cortex, which contain pyramidal neurons, but not in the dentate gyrus, which is composed primarily of granular cells, or the basal ganglia (Figure 1). We then examined the relationship between NMDAR density in the CA1 and the level of pathology (Braak stage). NMDAR density was significantly reduced in subjects with pathological findings indicative of definite AD (Braak stages V and VI) as compared to Braak stages 0-II and there was a linear inverse relationship between disease progression and NMDAR density. The presence of the APOE4 allele, a major genetic risk factor for AD [13] was also associated with decreased NMDAR (Figure 1). Significant inverse correlation was also observed in the CA3 field of the hippocampus and entorhinal cortex, but not in dentate gyrus or striatum (not shown).

We next examined the hypothesis that region-selective loss of NMDAR is related to neuroinflammation. It is now generally accepted that the neurofibrillary tangles (NFTs) and neuritic plaques characteristic of AD are associated with a host of inflammatory molecules, including complement proteins, as well as with activated microglia [14]. Both *in vivo* and postmortem studies have demonstrated increases in activated microglia in AD utilizing ligands for the neuroinflammation marker translocator protein 18kDA (TSPO, [7,15,19]), a mitochondrial membrane protein (formerly known as peripheral benzodiazepine receptor, [16]) with increased expression in activated microglia [17]. In animals, neuroinflammation induced by lipopolysaccharide (LPS) infusion reproduced components of the neurobiology and anatomy of Alzheimer’s disease, such as increase in beta amyloid precursor protein (βAPP) mRNA levels, degeneration of hippocampal pyramidal neurons, significant impairment in spatial memory and decrease on NMDAR density in hippocampus and temporal cortex, but not basal ganglia [19,22,23]. Our group also showed a reciprocal, region-dependent relationship between neuroinflammation and loss of NMDAR in other pathologies involving both neuroinflammation and cognitive loss, including animal models of brain injury and stroke [24-26].

**Figure 2:**
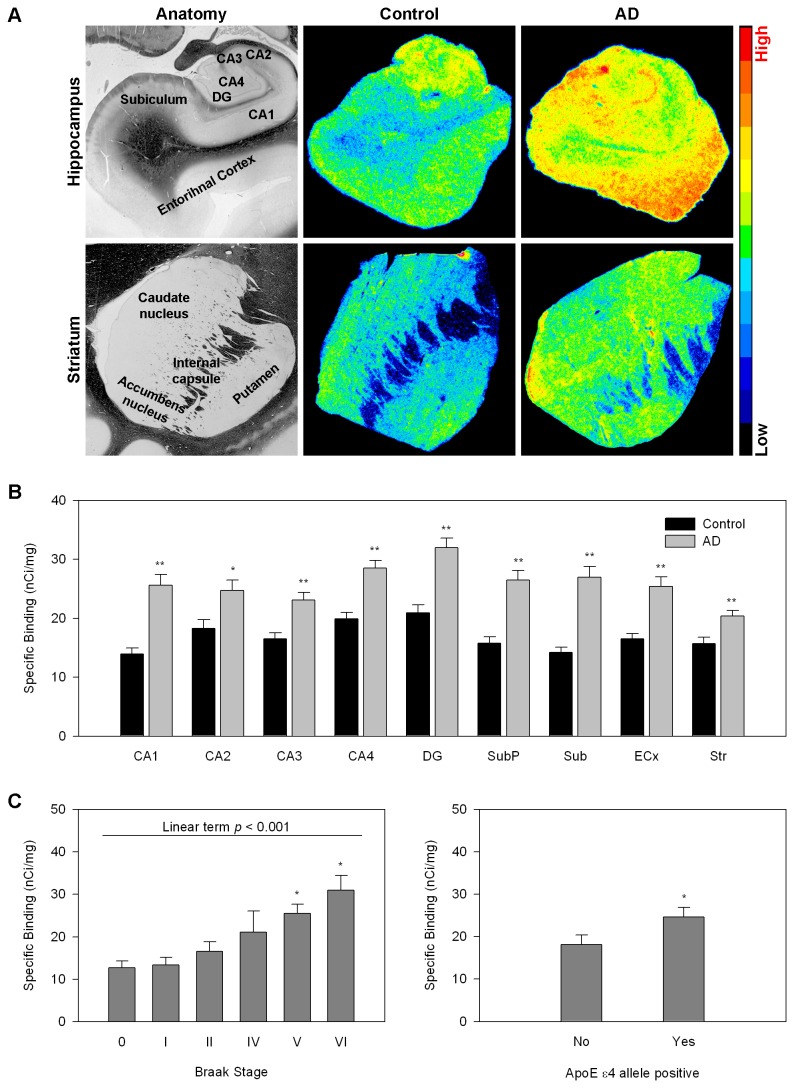
Regional neuroinflammation in AD subjects and controls measured with [^3^H]PK11195 autoradiography. A. Representative autoradiographic and anatomical images. Top –Hippocampal anatomy is shown on the left followed by a representative pseudocolored TSPO autoradiogram from hippocampus of a control subject and a representative autoradiogram from hippocampus of a subject with AD. The bottom row depicts striatal anatomy on the left followed by a representative TSPO autoradiogram from a control striatum and a representative autoradiogram from a subject with AD. Autoradiograms were pseudocolored using the rainbow spectrum (bar on the right). B. Quantitative autoradiographic measurements of regional TSPO density (expressed as specific binding of [3H]PK11195 in nCi/mg tissue). Abbreviations: CA1-4 cornu ammoni fields of the hippocampus, DG=gentate gyrus, SubP=subpyramidal layers of CA1, Sub=subiculum, ECx=entorhinal cortex, Str=striatum. *p<0.05, **p<0.005 AD relative to control; ANOVA followed by Fisher’s PLSD posthoc test. C. TSPO density in CA1 pyramidal cell body layer as a function of Braak stage (left) and genotype (right). *p<0.05 relative to Braak stages 0-II, one way ANOVA followed by Fisher’s PLSD posthoc test. **p<0.005, student’s t test.

**Figure 3:**
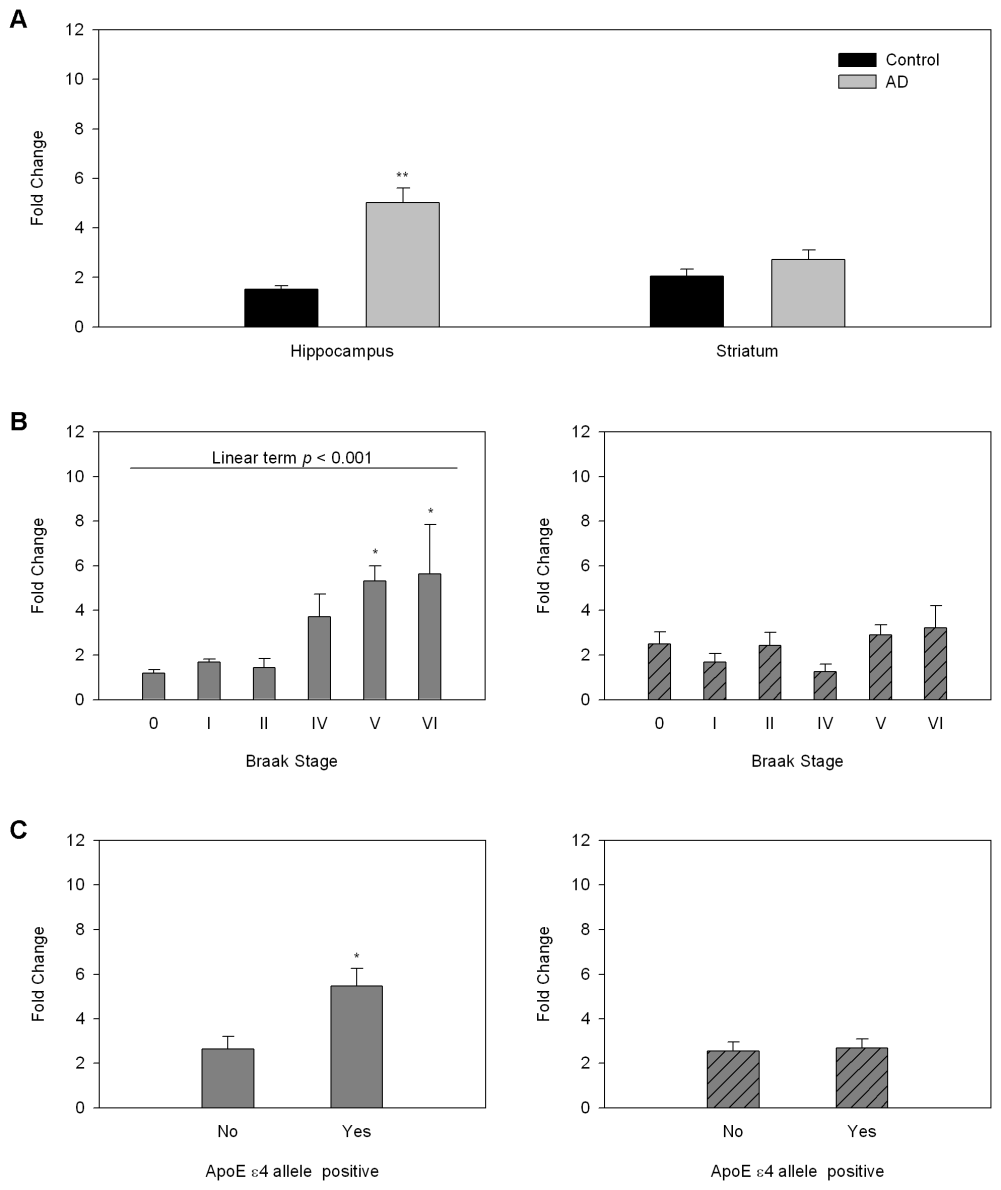
Regional TSPO mRNA gene expression in relation to AD pathology and ApoE genotype. A. TSPO gene expression by region and disease state. TSPO mRNA gene expression was measured by RT-PCR and expressed as fold change over expression in the reference sample. **p<0.005. B. TSPO gene expression as a function of Braak stage in hippocampus (left) and striatum (right). *p<0.05 relative to Braak stages 0-II, one way ANOVA followed by Fisher’s PLSD posthoc test. C. Effect of genotype on TSPO gene expression in hippocampus (left) and striatum (right). *p<0.05, students t-test 2 tailed.

We performed quantitative in vitro autoradiographic studies of the selective TSPO ligand [^3^H]PK11195 [17] on consecutive sections from the same brain samples in which we measured NMDAR density. As expected, we found significant increases in TSPO in all of the brain regions investigated (Figure 2). TSPO density in the CA1 field of the hippocampus was correlated with increased AD pathology and increased in the presence of the ApoE e4 allele (Figure 2). Similar correlations were seen in other hippocampal fields and in the entorhinal cortex but not in the basal ganglia (not shown). This pattern was seen also when we investigated TSPO mRNA gene expression in samples of hippocampus and striatum by real time PCR. Gene expression of TSPO was significantly increased in the hippocampus of AD patients while the increase in striatum did not reach statistical significance. Braak stage and the presence of the ApoE e4 allele were positively correlated with TSPO gene expression in hippocampus but not striatum (Figure 3). 

**Figure 4:**
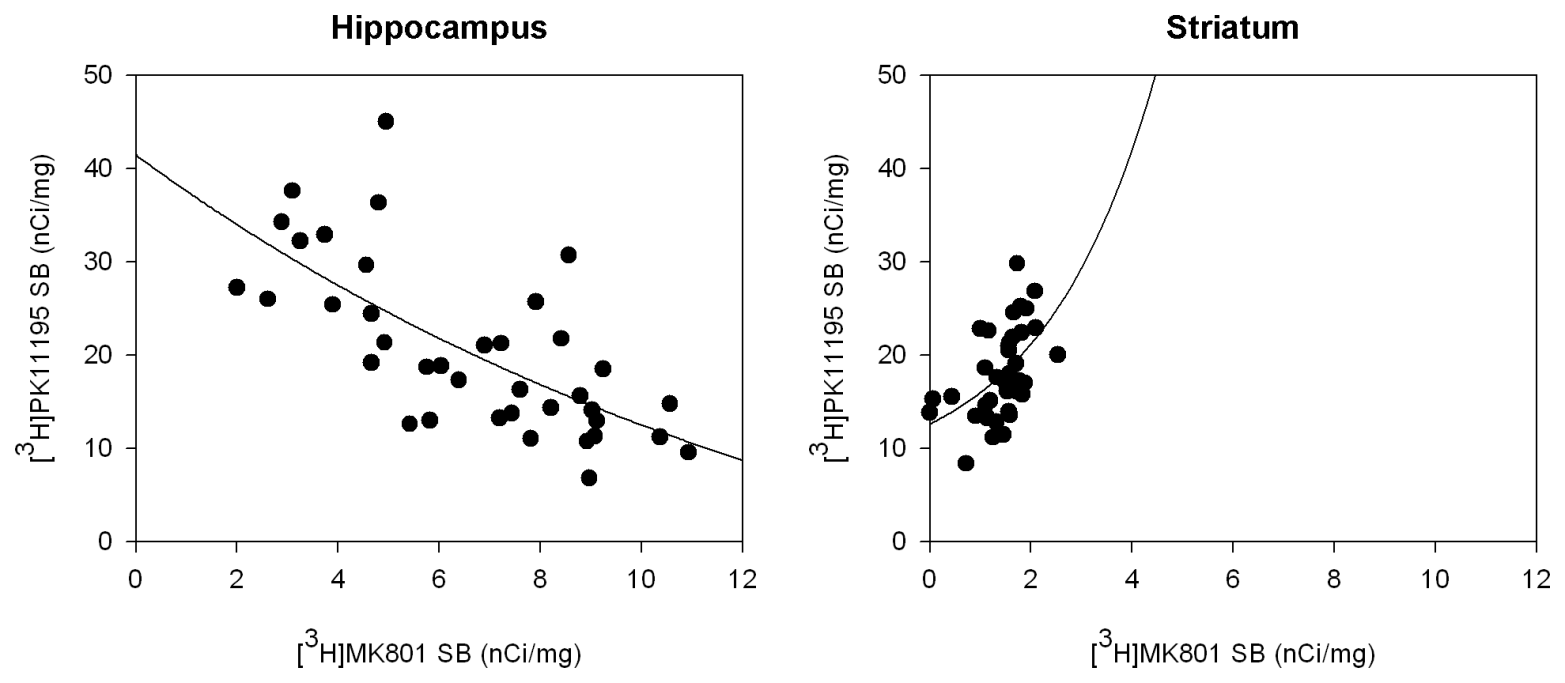
Region dependent correlation between TSPO and NMDAR density. Quantitative analyses of [^3^H]MK801 binding to NMDAR and [^3^H]PK11195 binding to TSPO were performed by autoradiography. The correlation between NMDAR and TSPO density was negative in the CA1 hippocampal field (left, *n* = 38, Spearman’s *p* < 0.001) and positive in the striatum (right, *n* = 37, Spearman’s *p* < 0.001). SB=specific binding.

Finally, we examined the regional relationship between NMDAR and TSPO densities measured in the same individuals (Figure 4). We found a highly significant negative correlation between TSPO and NMDAR in the hippocampal CA1 field. Conversely, TSPO density in basal ganglia was positively correlated with NMDAR density. These results suggest that the response to neuroinflammation is inherently different in hippocampus and basal ganglia and that hippocampal NMDAR expressing cells are preferentially vulnerable to neuroinflammation. Although the source of this qualitative difference has not been established in the human brain to date, we have made similar observations in rodent neuronal culture, where we have shown that the inflammatory cytokine TNF-alpha induces neurodegeneration in cortical cells while inducing neuroprotection of striatal neurons grown in the same culture. In the same experimental system, we have shown greater toxicity of glutamate on cortical cells relative to striatal cells, which was augmented by TNF-alpha in cortical cells and inhibited by TNFalpha in striatal cells [23]. 

Taken together, our results and the published observations summarized above support a pathway to symptom emergence and progression in AD whereby age-related accumulation of amyloid plaques throughout the brain [4] causes widespread neuorinflammation which is locally augmented by selective NFT deposition in hippocampus and entorhinal cortex [2]. This, in turn, leads to loss of NMDAR density and loss of NMDAR expressing cells [27] in these regions, followed by secondary neuroinflammation in response to neuronal loss and disease progression. This model is supported by the correlation between regional neuroinflammation, but not plaque load, and cognitive abilities [7,21]; the decreased risk of AD in users of non steroidal anti-inflammatory agents coupled with the lack of efficacy of the same drugs in symptomatic AD [28,29], evidence of neuroinflammation in subjects with mild cognitive impairment who later converted to dementia [30] and the efficacy of memantine, a low-affinity non-competitive NMDAR antagonist which protects physiological synaptic transmission through NMDAR, in moderate to severe AD [31]. Furthermore, the close association between the region-specific decrease in NMDAR density and the progression of definite AD supports the use of positron-emitting NMDAR antagonists such as [^11^C]CNS5161 [32,33] in the diagnosis and staging of AD *in vivo*. 

An NMDAR selective radiotracer may prove to be an important addition to other radiotracers used in PET studies of AD to date (e.g. [^18^F]FDG, [^11^C]PIB and [^11^C]PK11195, reviewed in [34]) facilitating *in vivo* diagnosis and staging of advanced AD as well as subject selection for treatment with drugs such as memantine, which target NMDAR.
